# High Phosphorus Diet-Induced Changes in NaPi-IIb Phosphate Transporter Expression in the Rat Kidney: DNA Microarray Analysis

**DOI:** 10.1371/journal.pone.0029483

**Published:** 2012-01-03

**Authors:** Tatsuya Suyama, Shinji Okada, Tomoko Ishijima, Kota Iida, Keiko Abe, Yuji Nakai

**Affiliations:** Graduate School of Agricultural and Life Sciences, The University of Tokyo, Tokyo, Japan; Louisiana State University and A & M College, United States of America

## Abstract

The mechanism by which phosphorus levels are maintained in the body was investigated by analyzing changes in gene expression in the rat kidney following administration of a high phosphorus (HP) diet. Male Wistar rats were divided into two groups and fed a diet containing 0.3% (control) or 1.2% (HP) phosphorous for 24 days. Phosphorous retention was not significantly increased in HP rats, but fractional excretion of phosphorus was significantly increased in the HP group compared to controls, with an excessive amount of the ingested phosphorus being passed through the body. DNA microarray analysis of kidney tissue from both groups revealed changes in gene expression profile induced by a HP diet. Among the genes that were upregulated, Gene Ontology (GO) terms related to ossification, collagen fibril organization, and inflammation and immune response were significantly enriched. In particular, there was significant upregulation of type IIb sodium-dependent phosphate transporter (NaPi-IIb) in the HP rat kidney compared to control rats. This upregulation was confirmed by *in situ* hybridization. Distinct signals for NaPi-IIb in both the cortex and medulla of the kidney were apparent in the HP group, while the corresponding signals were much weaker in the control group. Immunohistochemical analysis showed that NaPi-IIb localized to the basolateral side of kidney epithelial cells surrounding the urinary duct in HP rats but not in control animals. These data suggest that NaPi-IIb is upregulated in the kidney in response to the active excretion of phosphate in HP diet-fed rats.

## Introduction

Phosphorus is an important factor in numerous biological processes and exists in the form of inorganic phosphates in the body. The intake of dietary phosphate has been gradually increasing with changes in life style over the past several decades [1]. In healthy subjects, present-day levels of dietary phosphate are not likely to cause imminent hyperphosphatemia; however, excessive intake could present serious problems, particularly for chronic renal patients. Therefore, our understanding of the mechanisms of phosphate homeostasis is extremely important.

The major organs involved in phosphate homeostasis are the small intestine, kidney, parathyroid gland, and bone. Serum phosphate levels are tightly-regulated through the action of humoral factors such as parathyroid hormone (PTH), fibroblast growth factor 23, and 1α,25-dihydroxyvitamine D (also known as calcitriol). The expression or synthesis of these factors is coordinately regulated in response to changes in dietary and serum phosphate levels [2]. However, the mechanism of regulation of phosphate homeostasis, including effector molecules such as phosphate transporters, remains to be elucidated.

The kidney plays a pivotal role in phosphate excretion and has been the focus of many studies, particularly those studying the effects of a high phosphorous (HP) diet in experimental animals. Administration of a HP diet causes renal calcification and infiltration of inflammatory cells [3]. Furthermore, previous reports have suggested that there are major alterations in the mRNA expression profile in response to a HP diet, including downregulation of sodium-dependent phosphate transporter (NaPi)-IIa and NaPi-IIc [4], [5], and upregulation of osteopontin in the rat kidney [6]. However, relatively few studies have examined the influence of a HP diet on renal gene expression in a comprehensive manner. Dietary phosphate-responsive genes have been reported in rainbow trout kidney [7], but the global effects of a HP diet on mammalian gene expression in the kidney have yet to be reported.

To investigate the mechanism(s) by which phosphate levels are maintained in the body, gene expression in the kidney of HP diet-fed rats was assessed by DNA microarray analysis. The effect of administration of a HP diet on overall gene expression in the kidney as well as the induction of specific genes such as NaPi-IIb in response to a HP diet was demonstrated.

## Results

### Food intake and body weight

Food and calcium intake, body weight at baseline and study end, and average weight gain were not significantly different between control and HP-fed rats (Table 1). As expected, phosphorus intake, calculated from measured food intake and the known phosphorus content of the diet, was significantly higher in the HP group than in control animals (Table 2).

**Table 1:** Body weight, weight gain and food intake in control and HP rats.

	Control	HP
Initial body weight (g)	110.77±0.99	110.72±2.68
Final body weight (g)	243.38±4.67	236.75±5.31
Weight gain (g/day)	5.53±0.16	5.25±0.12
Food intake (g/day)	15.75±0.40	15.11±0.21

Data represent means ± SE (*n* = 5).

**Table 2:** Phosphorus and calcium balance and net absorption for control and HP diets.

		Control	High phosphorus
Serum concentration	Phosphorus (mg/dl)	7.70±0.21	7.11±0.13*
	Calcium (mg/dl)	9.56±0.08	9.47±0.09
	Urea nitrogen (mg/dl)	16.78±0.37	15.64±0.76
Phosphorus balance	Intake (mg/day)	53.94±1.81	197.95±3.51*
	Fecal (mg/day)	18.82±0.87	30.89±1.04*
	Urinary (mg/day)	4.68±1.10	133.11±3.08*
	Net absorption (mg/day)	35.13±1.39	167.06±2.62*
	Retention (mg/day)	30.45±0.74	33.95±2.53
Calcium balance	Intake (mg/day)	89.91±3.01	82.48±1.46
	Fecal (mg/day)	44.53±1.60	41.67±1.48
	Urinary (mg/day)	1.16±0.24	1.04±0.07
	Net absorption (mg/day)	45.37±1.82	40.81±1.17
	Retention (mg/day)	44.21±1.81	39.77±1.16

Data represent means ± SE (*n* = 5).

*p<0.05 compared to the control group.

Net absorption and retention were calculated as follows:

Net absorption (mg/day) = intake – fecal excretion.

Retention (mg/day) = net absorption – urinary excretion.

### Serum phosphorus, calcium and urea nitrogen levels

**Figure 1:**
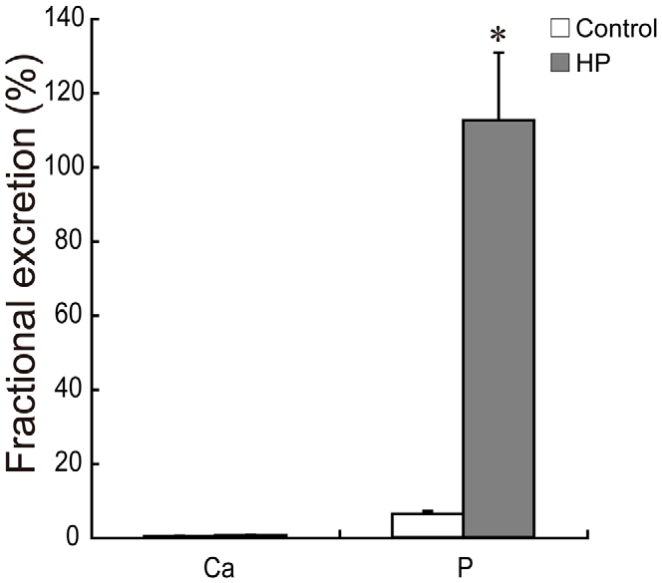
Effect of a HP diet on fractional excretion of calcium and phosphate in the kidney. Data represent means ± SE (*n* = 6 or 7). *p<0.05 compared to the control group.

HP rats had significantly lower serum phosphorus concentrations, while serum calcium concentrations were similar between the two groups (Table 2). To determine whether the intake of a HP diet affected renal function, blood urea nitrogen (BUN) was measured, which is an index of renal function [8]. BUN levels did not differ significantly between the two groups (Table 2). On the other hand, fractional excretion of phosphate was significantly increased in the HP group, while fractional excretion of calcium was similar (Fig. 1).

### Effects of a HP diet on phosphorus and calcium balance

Net intestinal phosphorus absorption was significantly increased in the HP group, whereas phosphorous retention between the two groups was similar (Table 2). Thus, an excessive amount of the ingested phosphorus was passed through the body in HP diet-fed animals. There were no significant differences in parameters of calcium balance between the two groups (Table 2).

### Kidney weight and mineral content

**Figure 2:**
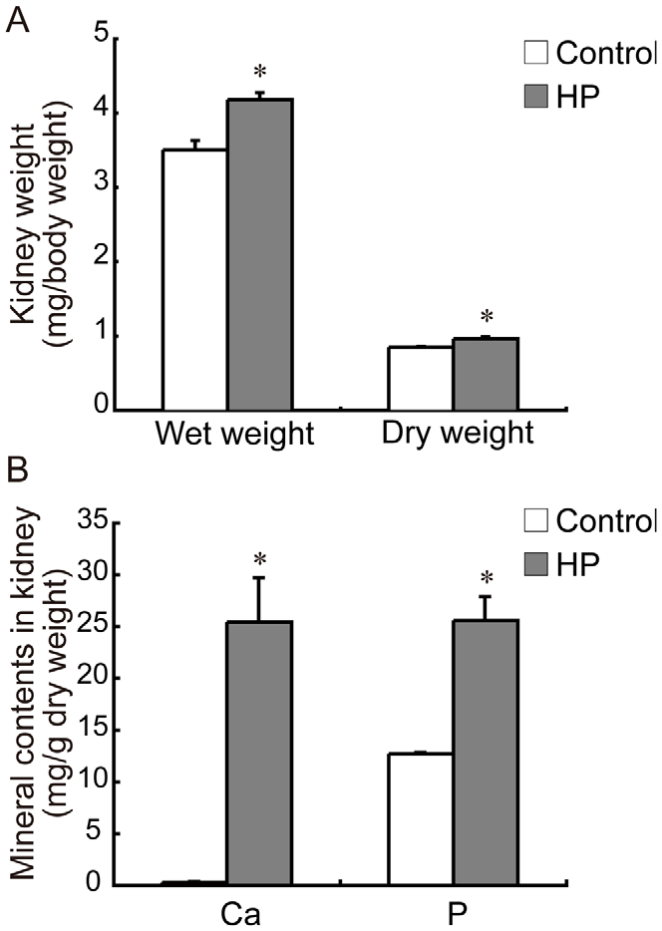
Effect of a HP diet on kidney weight and kidney mineral content. (A) Kidney weight according to body weight; (B) Kidney mineral content per dry weight of kidney. Data represent means ± SE (*n* = 5). *p<0.05 compared to the control group.

Kidney weight, both wet and dry weight, was significantly increased in the HP group (Fig. 2A). Kidney calcium and phosphorus content was also significantly increased in the HP group compared to controls (Fig. 2B). These results suggested that there was some degree of calcification and hypertrophy of the kidney in HP diet-fed rats.

### Histological analysis

**Figure 3:**
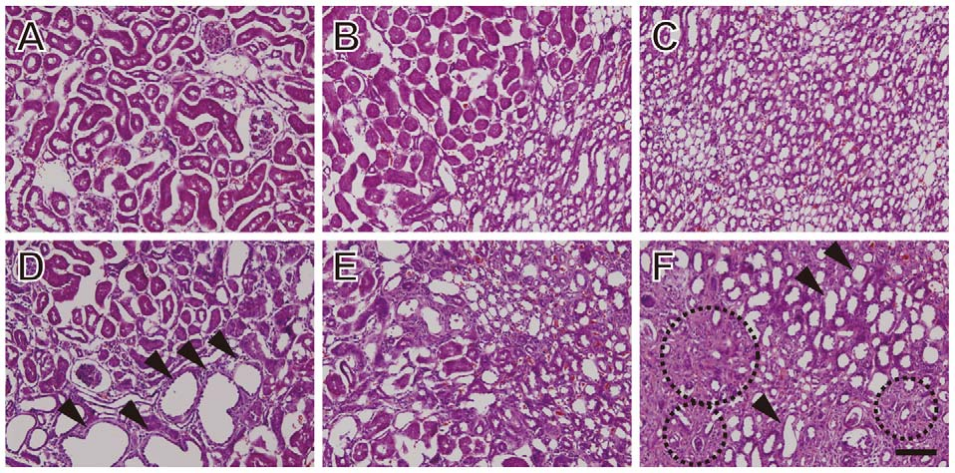
Histological analysis of kidney tissue sections from rats fed a control or HP diet. Kidney sections from control rats (A, B, C) and HP rats (D, E, F) were stained with H&E and visualized by microscopy. Sections correspond to cortex (A, D), the corticomedullary junction (B, E) and the medullary region (C, F). Arrowheads indicate representative cyst-like areas; dotted circles indicate representative fibrosis-like areas. Scale bar = 100 µm.

To evaluate the effect of a HP diet on kidney morphology, histology was performed on kidney tissue sections using hematoxylin and eosin (H&E) staining (Fig. 3A–F). In rats fed a HP diet, cyst-like swelling was apparent in the renal cortex (Fig. 3D) and medulla (Fig. 3F) of the kidney. Fibril formation was also noted, especially in the renal medulla (Fig. 3F). These results suggested that intake of a HP diet induces morphological abnormalities in the kidney.

### DNA microarray analysis of gene expression

The distribution free weighted method (DFW) [9] quantified microarray data were subjected to hierarchical clustering analysis. Each experimental group formed a large and separate cluster (Fig. S1), which indicated that a HP diet can induce changes in gene expression profile in the rat kidney.

To identify differentially expressed genes (DEGs) in response to a HP diet, the rank products (RP) method [10] was applied to DFW-quantified data. The RP method combined with a DFW preprocessing algorithm has been shown to be one of the best ways to accurately detect DEGs [11]. Applying a significance value for the false discovery rate (FDR) of <0.05, we identified 1056 upregulated probe sets (838 genes) and 712 downregulated probe sets (536 genes) in the HP group compared to the control group. The full list of DEGs is shown in Table S1.

### Gene Ontology (GO) analysis

DEGs were classified into functional categories according to GO. GO terms that were significantly enriched within the two sets of DEGs (upregulated and downregulated genes) are summarized in Tables 3 and 4, respectively. The overrepresented GO terms in the upregulated genes were further classified into three major GO term clusters: “ossification”, “collagen fibril organization” and diverse clusters of “inflammatory and immune response” (Table 3).

**Table 3:** GO terms that were significantly enriched (*p*<0.05) in the top 1056 genes upregulated in response to a HP diet.

GO-ID	GO term	FDR-corrected *p*-value
0032502	Developmental process*	
0002520	Immune system development	**1.30E-04** **
0001944	Vasculature development	1.38E-02
0001568	Blood vessel development	**1.03E-02**
0001501	Skeletal system development	2.70E-03
0001503	Ossification	4.37E-03
0001957	Intramembranous ossification	**4.23E-02**
0009987	Cellular process	
0001558	Regulation of cell growth	**1.42E-02**
0001775	Cell activation	1.17E-12
0002274	Myeloid leukocyte activation	**1.92E-02**
0043062	Extracellular structure organization	2.41E-07
0030199	Collagen fibril organization	**9.56E-05**
0007155	Cell adhesion	3.09E-05
0001953	Negative regulation of cell-matrix adhesion	**2.07E-02**
0032501	Multicellular organismal process	
0001819	Positive regulation of cytokine production	**9.94E-03**
0042221	Response to chemical stimulus	
0000302	Response to reactive oxygen species	**1.25E-02**
0002237	Response to molecule of bacterial origin	**5.51E-04**
0002376	Immune system process	
0006955	Immune response	7.27E-20
0002252	Immune effector process	5.15E-09
0001910	Regulation of leukocyte mediated cytotoxicity	6.15E-04
0001911	Negative regulation of leukocyte mediated cytotoxicity	**1.82E-03**
0001914	Regulation of T cell mediated cytotoxicity	7.24E-04
0001916	Positive regulation of T cell mediated cytotoxicity	**1.38E-02**
0050778	Positive regulation of immune response	2.85E-13
0002253	Activation of immune response	**2.62E-07**
0002675	Positive regulation of acute inflammatory	2.68E-03
0002885	Positive regulation of hypersensitivity	4.23E-02
0001798	Positive regulation of type IIa hypersensitivity	**2.10E-02**

*GO term with no *p*-value means not significant.

**FDR-corrected *p*-values of the GO terms appearing in the deepest hierarchy are represented by bold style.

**Table 4:** GO terms that were significantly enriched (*p*<0.05) in the top 712 genes downregulated in response to a HP diet.

GO-ID	GO term	FDR-corrected *p*-value
0008152	Metabolic process*	
0055114	Oxidation reduction	**3.09E-03** **
0016053	Organic acid biosynthetic process	9.76E-04
0046394	Carboxylic acid biosynthetic process	**9.76E-04**
0050896	Response to stimulus	
0007584	Response to nutrient	**1.02E-02**

*GO term with no *p*-value means not significant.

**FDR-corrected *p*-values of the GO terms appearing in the deepest hierarchy are represented by bold style.

Among the DEGs related to ossification we found secreted phosphoprotein 1 (SPP1; also known as osteopontin). This result was in good agreement with a previous study showing a marked induction of SPP1 in response to a HP diet [6]. Also identified were genes for non-collagenous bone matrix proteins including glycoprotein (transmembrane) nmb (Gpnmb/osteoactivin), secreted protein acidic and rich in cysteine (SPARC/osteonectin), and fibronectin 1 (Fn1). The GO term ossification also included genes for several collagens such as collagen type I alpha 1 (Col1a1), type V alpha 2 (Col5a2), and type XI alpha 1 (Col11a1), as well as humoral factors such as bone morphogenetic protein (Bmp6) and stanniocalcin 1 (Stc1). Within the GO term collagen fibril organization were a number of genes encoding fibrous collagen proteins, including collagen type I alpha 2 (Col1a2), type III alpha 1 (Col3a1), type V alpha 1 (Col5a1) and alpha 2 (Col5a2), all of which are components of the extracellular matrix. Transforming growth factor beta 2 (TGFb2) was also included within this term.

The third GO cluster in the upregulated genes included many genes related to inflammatory or immune responses. Quick GO analysis revealed that these genes were strongly associated with two GO terms, “positive regulation of Type IIa hypersensitivity” and “positive regulation of T cell mediated cytotoxicity”, and both groups included genes encoding complement component 3 (C3), Fc receptors and major histocompatibility complex (MHC) class I molecules.

Downregulated genes in HP rats included three overrepresented GO terms, “oxidation reduction”, “carboxylic acid biosynthetic process” and “response to nutrient” (Table 4). The genes included within these GO terms are listed in Table S2.

### Changes in gene expression of transporter or channel for phosphate, calcium and water

Gene-annotation enrichment analysis (as above) is able to detect only those genes with the same GO annotation that are statistically enriched in a given population of DEGs. To complement the GO analysis, we selected DEGs related to transporters or channels for phosphate, water and calcium for further analysis (Table 5). Among known phosphate transporters, solute carrier family 34 (Slc34a) members 1, 2 and 3 (Slc34a1/2/3), also known as type II sodium-dependent phosphate cotransporters (NaPi), were differentially expressed (NaPi-IIa and -IIc were downregulated, while NaPi-IIb was upregulated) in response to a HP diet. Aquaporin11 (Aqp11), a water channel, was downregulated in the kidney in HP rats. Furthermore, differentially expressed genes for calcium channels and transporters were as follows: ATPase Ca^2+^ transporting plasma membrane 1 (Atp2b1), type 2C member 2 (Atp2c2), Calcium channel voltage- dependent T type alpha 1I subunit (Cacna1i), and alpha 2/delta subunit 1 (Cacna2d1). However, these four genes were only small part of many calcium channels and transporters. In fact, there were no significant differences in fractional excretion of calcium between the HP and control groups, it seems likely that these four genes are not critical for calcium reabsorption and secretion in kidney.

**Table 5:** Genes related to water, phosphorus and calcium transport whose expression was altered in response to a HP diet.

	Probe set ID	Gene symbol	Gene title	Gene expression
Water	1384877_at	Aqp11	Aquaporin 11	Down
Phosphorus	1370610_at	Slc34a1	Solute carrier family 34 (sodium phosphate), member 1 (NaPi-IIa)	Down
	1368168_at	Slc34a2	Solute carrier family 34 (sodium phosphate), member 2 (NaPi-IIb)	Up
	1384838_at	Slc34a3	Solute carrier family 34 (sodium phosphate), member 3 (NaPi-IIc)	Down
Calcium	1394714_at	Atp2b1	ATPase, Ca^2+^ transporting, plasma membrane 1	Up
	1387310_at	Atp2c2	ATPase, Ca^2+^ transporting, type 2C, member 2	Down
	1369211_at	Cacna1i	Calcium channel, voltage-dependent, T type, alpha 1I subunit	Down
	1369649_at	Cacna2d1	Calcium channel, voltage-dependent, alpha2/delta subunit 1	Up

### *In situ* hybridization analysis of NaPi-IIb in the rat kidney

**Figure 4:**
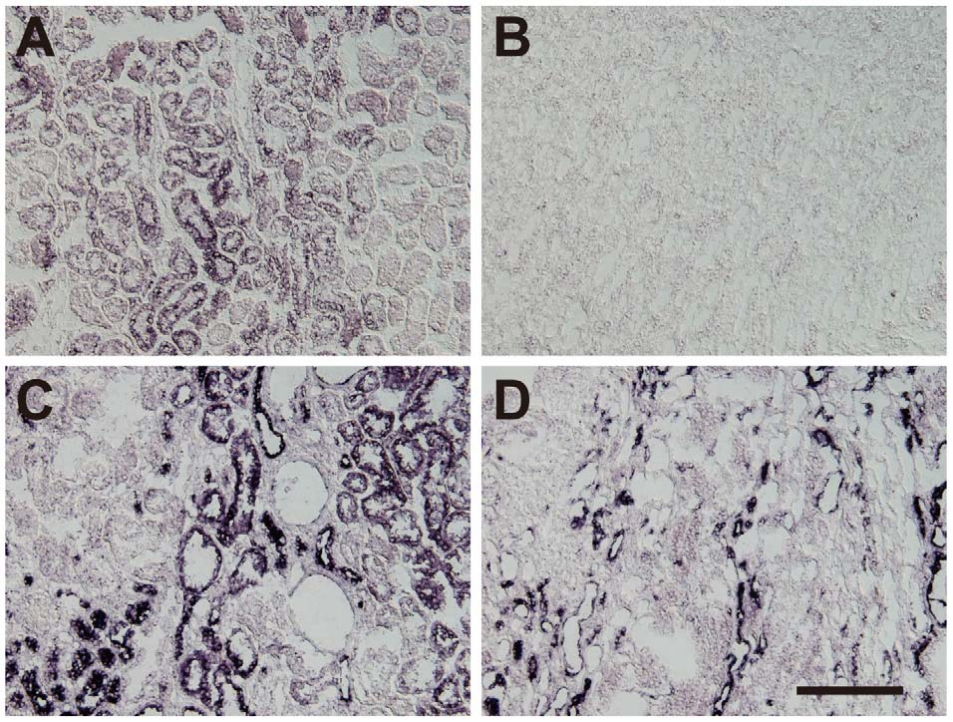
Expression of NaPi-IIb in the kidney in control and HP rats. Kidney sections from control (A, B) and HP (C, D) rats were analyzed by *in situ* hybridization using a NaPi-IIb-specific probe. Sections represent the cortex (A, C) and medulla (B, D). Scale bar = 200 µm.

*In situ* hybridization was carried out to confirm the differential expression of Slc34 family mRNAs in the rat kidney in response to a HP diet (Fig. 4). Weak NaPi-IIb-positive signals were observed in the cortex (Fig. 4A) but not the medulla (Fig. 4B) of the kidney in the control group. In contrast, the corresponding signals clearly localized to the kidney cortex (Fig. 4C) and medulla (Fig. 4D) in the HP group. No signals were detectable in the negative control samples (data not shown). The downregulation of NaPi-IIa mRNA expression was also confirmed by *in situ* hybridization (data not shown).

### Immunohistochemical analysis of NaPi-IIb in the rat kidney

**Figure 5:**
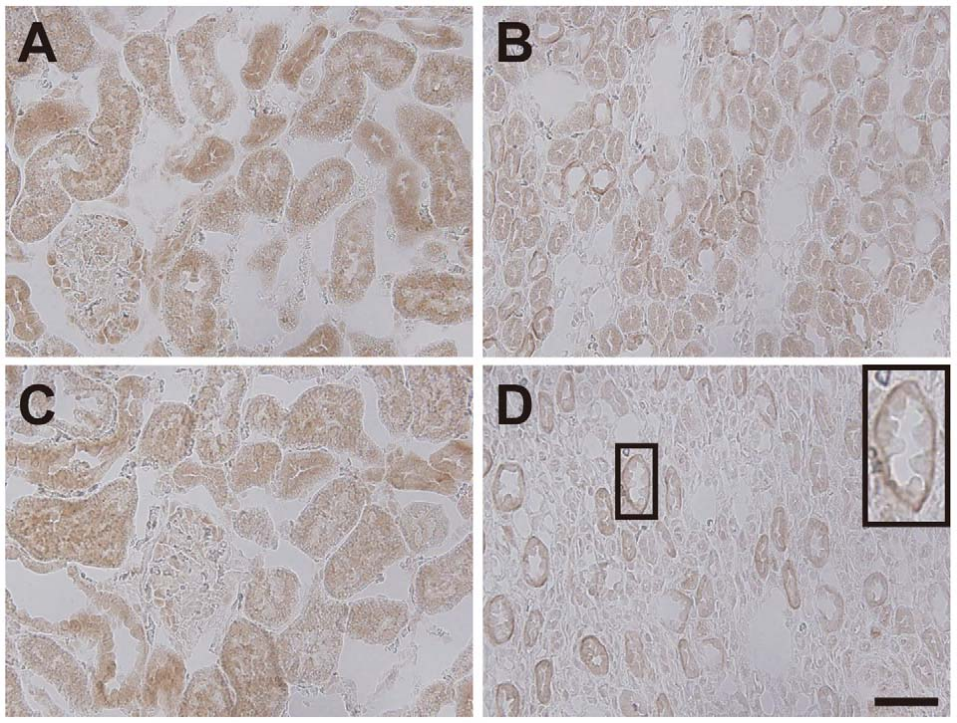
Immunohistochemical analysis of NaPi-IIb in the kidney of control and HP rats. Tissue sections from control (A, B) and HP (C, D) rats were analyzed by immunohistochemistry using an anti-NaPi-IIb antibody. Sections represent cortex (A, C) and the medullary regions (B, D). Scale bar = 50 µm.

To determine whether NaPi-IIb localization exhibited any polarity, kidney tissue sections were analyzed by immunohistochemistry using an anti-NaPi-IIb antibody (Fig. 5). NaPi-IIb localized predominantly to the cytosol in the cortex (Fig. 5A) and medulla (Fig. 5B) of the kidney in the control group. In the HP group, NaPi-IIb also localized to the cytosol in the cortex (Fig. 5C), whereas in the medulla, NaPi-IIb was located at the basolateral membrane of the epithelial cells surrounding the urinary duct (Fig. 5D).

## Discussion

DNA microarray analysis was used to investigate the effects of a HP diet on gene expression in the rat kidney. HP rats were administered a diet containing 1.2% phosphorus, which was 4-times the amount in the AIN-93G control diet, for 24 days. Under these conditions, there were no significant differences between control and HP rats in terms of final body weight and food intake. Apparent phosphorus absorption as well as urinary and fecal phosphorus excretion was significantly increased in the HP group, whereas there was no significant difference in phosphorus retention between the two groups. These results suggest that a large amount of the phosphorus that was ingested in the HP group passed through the body.

Serum phosphorous concentrations were significantly lower in the HP group compared to the control group. This discrepancy may be due to the timing of blood sampling, and the fact that the fractional excretion of phosphorus was increased in the HP group. Rats were sacrificed after a 4 h fast, during which there was no further ingestion of phosphorus. Thus, during this period, blood phosphate was being actively excreted, which could lead to lower serum concentrations in the HP group than in the control animals. In a previous study, there was a negative phosphorus balance in rats given a 1.2% or 1.5% phosphorus diet [4], supporting the idea that enhanced excretion of phosphorus occurs as a result of ingestion of a HP diet.

There was a significant increase in kidney weight in the HP group, indicating renal hypertrophy, and significant increases in the phosphorus and calcium content of the kidney, indicating nephrocalcinosis. These results suggest that an excess amount of phosphorus passing through the body might be sufficient to induce a high-phosphorus phenotype, even though phosphorous retention and serum concentrations were not elevated in the HP group.

Hierarchical clustering analysis of DNA microarray data generated using mRNA isolated from control and HP rats revealed that there were marked differences in the gene expression profile of the kidney between the two groups. GO terms that were significantly enriched in the set of upregulated genes fell into three categories: ossification, collagen fibril organization, and inflammatory (or immune) response. Nephrocalcinosis, fibrosis and inflammation are major symptoms induced in the kidney by a HP diet [3]. In fact, phosphorus and calcium content was increased in the kidney in the HP group, and histological analysis by H&E staining revealed fibrosis-like regions in kidney sections from HP rats. Thus, the microarray data and gene expression changes correlated with morphological and biochemical phenotypes in rats fed a HP diet.

Among the DEGs that mapped to the GO term ossification, we found a member of TGFβ superfamily gene, Bmp6. Previously, it was shown that exogenous Bmp6 induces the upregulation of a set of osteoblast-related genes, including SPP1, in human mesenchymal stem cells [12]. In light of these previous results, the data from the current study suggest a mechanism in which the upregulation of Bmp6 induces the differentiation of osteoblast-like cells in the kidney, resulting in the progression of nephrocalcinosis.

The GO term collagen fibril organization included Col1a2, Col3a1, Col5a1, Col5a2, and TGFb2. Previous reports have shown a transient upregulation of TGFb2 in acute-phase experimental nephritis, and this change in expression correlated with fibrosis progression [13], [14]. In a separate study, the addition of exogenous TGFb2 resulted in strong upregulation of Col1 and Col3 in keloid and burn hypertrophic scars [15]. Thus, upregulation of TGFb2 in response to a HP diet may induce the expression of extracellular matrix proteins that contribute to the fibril formation observed in the kidney medulla.

Many genes related to inflammatory or immune responses were differentially expressed in response to a HP diet. Quick GO analysis revealed that these genes mapped overwhelmingly to two GO terms, positive regulation of Type IIa hypersensitivity and positive regulation of T cell mediated cytotoxicity. These two categories included genes encoding C3, Fc receptors and MHC class I molecules. These results indicate that in HP rats, the complement pathway might be activated by IgG or IgM binding to sites in the kidney, leading to subsequent cell lysis or tissue damage by phagocytic cells such as macrophages or neutrophils (i.e. type II allergy-like immunoreactions). The upregulation of genes encoding MHC class I molecules also suggests the activation of macrophage or neutrophil chemotaxis in response to a HP diet. Col4 is a major component of the renal basement membrane and contains epitopes that trigger autoantibody formation [16]. Some chronic renal diseases are known to be caused by Col4 protein-autoantibody immune complexes [17]. The current results showing that Col4 was upregulated in response to a HP diet are consistent with these observations.

From the full set of DEGs, genes related to phosphate, calcium and water transport were extracted. Aquaporins (Aqps) are known to play major roles in water transport. There are 13 mammalian Aqps identified to date [18], [19]. Of these, only one, Aqp11, was altered (downregulated) by a HP diet; the expression levels of the others did not change significantly. Aqp11 is associated with polycystic renal disease and Aqp11 knockout animals develop cysts in the kidney [20]. We observed cyst-like swellings in kidney sections from the HP group, indicating that significant downregulation of Aqp11 may play a role in cyst formation in rats fed a HP diet.

Of the many calcium transporters or channels that have been identified, only 4 genes appeared to be differentially expressed in response to a HP diet: Atp2b1, Atp2c2, Cacna1i and Cacna2d1. However, since fractional excretion of calcium was not significantly affected by a HP diet, it is likely that these 4 genes are not involved in calcium homeostasis, particularly calcium excretion.

The three types of NaPi have been identified, NaPi-I, II and III corresponding to genes encoding Slc17, Slc34, and Slc20 solute carrier families, respectively [21]. NaPi-IIa and -IIc have been shown to be important for phosphate homeostasis [22]. They are expressed on the apical side of proximal tubule epithelial cells and play a pivotal role in phosphate reabsorption in the kidney [23]. NaPi-IIa and -IIc are downregulated in response to increased levels of dietary phosphate and serum PTH [4], [5], [24]. Consistent with these observations, we found that NaPi-IIa and -IIc were downregulated in response to high dietary intake of phosphorous in rats.

On the other hand, NaPi-IIb (Slc34a2) was significantly upregulated in HP rats. This upregulation of NaPi-IIb was confirmed by *in situ* hybridization, with marked expression observed in medullary epithelial cells. Immunohistochemical analysis showed that NaPi-IIb localized to the basolateral membrane of cells lining the collecting duct and/or the ascending limb of the loop of Henle. This cellular location of NaPi-IIb implies that it has a role in phosphate homeostasis, particularly excretion. Fractional excretion of phosphate was significantly increased in the HP group, with values exceeding 100%. This increase in phosphate excretion could not be explained solely by the downregulation of NaPi-IIa and -IIc. Thus, there appears to be other active excretion mechanism at play under conditions of a HP diet. The current data suggest that NaPi-IIb is upregulated in response to high dietary intake of phosphorous and results in enhanced phosphate excretion.

NaPi-IIb is expressed in the intestine, liver, epididymis and various other tissues, including the kidney [25]–[28]. A previous study using a conditional knockout approach demonstrated that NaPi-IIb plays a major role in phosphate absorption in the intestine [25]. Moreover, in mice, deletion of NaPi-IIb results in embryonic lethality [29], suggesting that NaPi-IIb is essential for survival. The function of NaPi-IIb in the kidney, however, remains to be fully elucidated.

Apical expression of NaPi-IIb has been demonstrated in the intestine [26], whereas in the kidney, the current data indicate that NaPi-IIb is expressed at the basolateral side of renal epithelial cells. From a broad localization standpoint, these results are not necessarily contradictory; in both cases, phosphate was transported from the outside of the cell to the interior. Thus, in HP rats, upregulation of NaPi-IIb can be thought of as enhancing basal phosphate excretion levels in the kidney. The other cotransporters NaPi-IIa and -IIc were shown to express on apical side of kidney epithelial cells both *in vivo* and *in vitro* [23], [24], [30]. These reports support that the role of NaPi-IIa and -IIc are exclusively phosphate reabsorption. On the other hand, under normal phosphate concentration condition, NaPi-IIb was shown to express on both apical and basolateral side of kidney epithelial cells *in vitro* [30]. While additional experiments using cultured kidney cells (MDCK or opossum kidney cells) will be needed especially under high phosphate conditions to define the events involved in phosphate transport, as well as the mechanisms underlying the polarity of NaPi-IIb expression, we are convinced that the current findings uncover a new facet of the mechanism of phosphate homeostasis.

## Materials and Methods

### Animals and diets

Male Wistar rats (4 weeks old) were purchased from Japan SLC Co. (Hamamatsu, Japan) and individually housed in metabolic cages under controlled conditions of 22±1°C and a 12-hour light/dark cycle (lights on from 08:00 to 20:00 daily). Two different diets containing 0.3% phosphorous (control diet) and 1.2% phosphorous (HP diet) based on the AIN-93G diet [31] (Table 6) were purchased from Research Diets Inc. (New Brunswick, NJ, USA). All rats were fed the control diet for a 7-day acclimatization period. After acclimatization, rats were divided into two groups of similar mean body weight (n = 5 each) and then fed either the control or the HP diet for 24 days. The animals were allowed to eat *ad libitum* and had free access to water (MilliQ water). Urine and feces were collected from day 20 to 23 for balance studies. At the end of the experimental period, all rats were sacrificed under anesthesia and blood and kidney samples were taken for analysis. Serum and urine samples were stored at −20°C until use. The right kidney was used for mineral analysis and the left kidney was used for DNA microarray analysis. For fractional excretion and histochemical analyses, an additional animal experiment was performed (n = 6 or 7) as described above except that the urine was collected on the last day and the left kidney was removed and sectioned. Tissue samples were frozen in liquid nitrogen immediately after excision and stored at −80°C until use. To measure calcium and phosphorus content, feces and kidney tissue were dried, ashed and then demineralized with a solution of HNO_3_ (0.1 mol/l). Calcium concentration was analyzed by SPS 1200VR Inductively Coupled Plasma (ICP)-Atomic Emission Spectrometry (Seiko Instruments Inc., Chiba, Japan). Phosphorus, BUN and creatinine were assayed using Phosphor C, Urea N B, and LabAssay™ Creatinine kits (Wako Pure Chemical Industries, Osaka, Japan), respectively. The protocol for the animal experiments was approved by the Animal Use Committee of the Faculty of Agriculture at The University of Tokyo (approval number: P09-283).

**Table 6:** Composition of the experimental diets.

	Control diet	HP diet
Ca level (%)	0.5	0.5
P level (%)	0.3	1.2
	g/kg diet	g/kg diet
Casein	200.0	200.0
Corn Starch	397.486	357.936
Maltodextrin 10	132.0	132.0
Sucrose	100.0	100.0
Soybean Oil	70.0	70.0
Cellulose	50.0	50.0
Mineral Mix	35.0	35.0
Vitamin Mix	10.0	10.0
L-Cystine	3.0	3.0
Choline Bitartrate	2.5	2.5
*t*-Butylhydoroquinone	0.014	0.014
KH_2_PO_4_	-	39.55

### DNA microarray experiments

Total RNA was isolated from the kidney using the TRIzol reagent (Invitrogen Life Technologies, Carlsbad, CA) and then purified using an RNeasy mini kit (Qiagen K.K., Tokyo, Japan). The quality and quantity of purified total RNA were verified by agarose gel electrophoresis and spectrophotometry, respectively. We selected four average rats from each group, according to determined by urine volume (vs. control group) and kidney phosphorus content (vs. HP group). DNA microarray analysis was carried out as described previously [32] with slight modifications. In brief, biotinylated aRNA was obtained from 100 ng of purified total RNA using a GeneChip 3′ IVT Express Kit (Affymetrix, Santa Clara, CA, USA). The aRNA was purified, fragmented and then hybridized to an Affymetrix Rat Genome 230 2.0 Array containing probe sets for over 30,000 rat genes. Following hybridization at 45°C for 16 h, the arrays were washed and labeled with phycoerythrin. Fluorescence signals were scanned using the Affymetrix GeneChip System. Affymetrix GeneChip Command Console software was used to reduce the array images to the intensity of each probe (CEL files). All the microarray data are MIAME compliant and have been deposited in a MIAME compliant database, the National Center for Biotechnology Information (NCBI) Gene Expression Omnibus (http://www.ncbi.nlm.nih.gov/geo/, GEO Series accession number GSE31973), as detailed on the MGED Society website (http://www.mged.org/Workgroups/MIAME/miame.html).

### DNA microarray data analysis

The CEL files were quantified with the DFW [9] using statistical language R (http://www.r-project) [33] and Bioconductor (http://www.bioconductor.org/) [34]. Hierarchical clustering was performed using the pvclust() function [35] in R. To identify DEGs, the RP method [10] was applied to DFW quantified data, with the number of permutations set at 1,000. Probe sets with an FDR <0.05 were regarded as having different expression levels between the two groups (i.e. were differently expressed). The annotation file for the Rat Genome 230 2.0 Array was downloaded from the Affymetrix web site (August 10, 2010, Rat230_2.na31.annot.csv).

Gene-annotation enrichment analysis of DEGs was performed using the Database for Annotation, Visualization and Integrated Discovery (DAVID; http://david.abcc.ncifcrf.gov/) [36] and Quick GO (http://www.ebi.ac.uk/QuickGO/) [37]. EASE scores, which are modified Fisher's exact test *p*-values [38], were used to extract statistically overrepresented GO terms from the DEGs; Benjamini and Hochberg FDR corrections [39] were used to correct the results by multiple testing. GO terms with FDR-corrected *p*-values of <0.05 were regarded as significantly enriched.

### In situ hybridization

Kidney tissue from control and HP rats was embedded in Tissue-Tek O. C. T compound (Sakura Finetechnical, Tokyo, Japan) and frozen in liquid nitrogen immediately after excision. Frozen tissue samples were sectioned into 7 µm-thick slices. The sections were fixed with paraformaldehyde (PFA), carboethoxylated and then treated with 10 µg/ml proteinase K for 30 min, followed by 4% PFA in PBS for 10 min. Sections were subjected to hybridization with riboprobes in hybridization buffer. The antisense riboprobes were synthesized from DNA fragments subcloned into pBluescript II SK(−) (Stratagene, LA Jolla, CA, USA) and hydrolyzed to approximately 500 bases before hybridization. As a negative control, *in situ* hybridization was performed using sense riboprobes.

### Immunohistochemistry

PFA-prefixed cryosections were prepared as described for *in situ* hybridization. Tissue sections were treated with Target Retrieval Solution (Dako, Carpinteria, CA, USA) for 20 min at 95°C and then cooled for 20 min at room temperature. After the inactivation of endogenous peroxidase with PBS containing 0.3% hydrogen peroxide for 30 min at room temperature, the sections were blocked with normal horse serum in PBS containing 0.1% Triton X-100 for 30 min, and then incubated at 4°C overnight with 3 µg/ml of rabbit anti-mouse NPT2b IgG (Alpha Diagnostic Intl. Inc., San Antonio, TX, USA). The sections were then incubated with ImmPRESS™ Reagent Horse Anti- Rabbit IgG (Vector Laboratories, Burlingame, CA, USA) for 30 min at room temperature. Visualization was performed with DAB Metal Enhanced Substrate kit (Pierce Biotechnology, Rockford, IL, USA).

## Supporting Information

Figure S1 Hierarchical clustering dendrograms from DFW-quantified DNA microarray data. High_P, high phosphorus diet group. Numbers represent independent samples. The vertical scale represents between-cluster distances.
(TIF)

Table S1 A full list of DEGs.
(XLS)

Table S2 Summary of GO terms majorly overrepresented within up- or downregulated genes in the HP diet group. The Table lists significantly enriched GO terms in DEGs. In the first sheet, genes included in each GO term are represented as gene symbols. Details of each GO term are described in individual sheets.
(XLS)
