# Supplementary figures and images for: High Phosphorus Diet-Induced Changes in NaPi-IIb Phosphate Transporter Expression in the Rat Kidney: DNA Microarray Analysis
Source: PLoS One. 2012 Jan 3;7(1):e29483. doi: 10.1371/journal.pone.0029483 (PMC3250443; doi:10.1371/journal.pone.0029483)

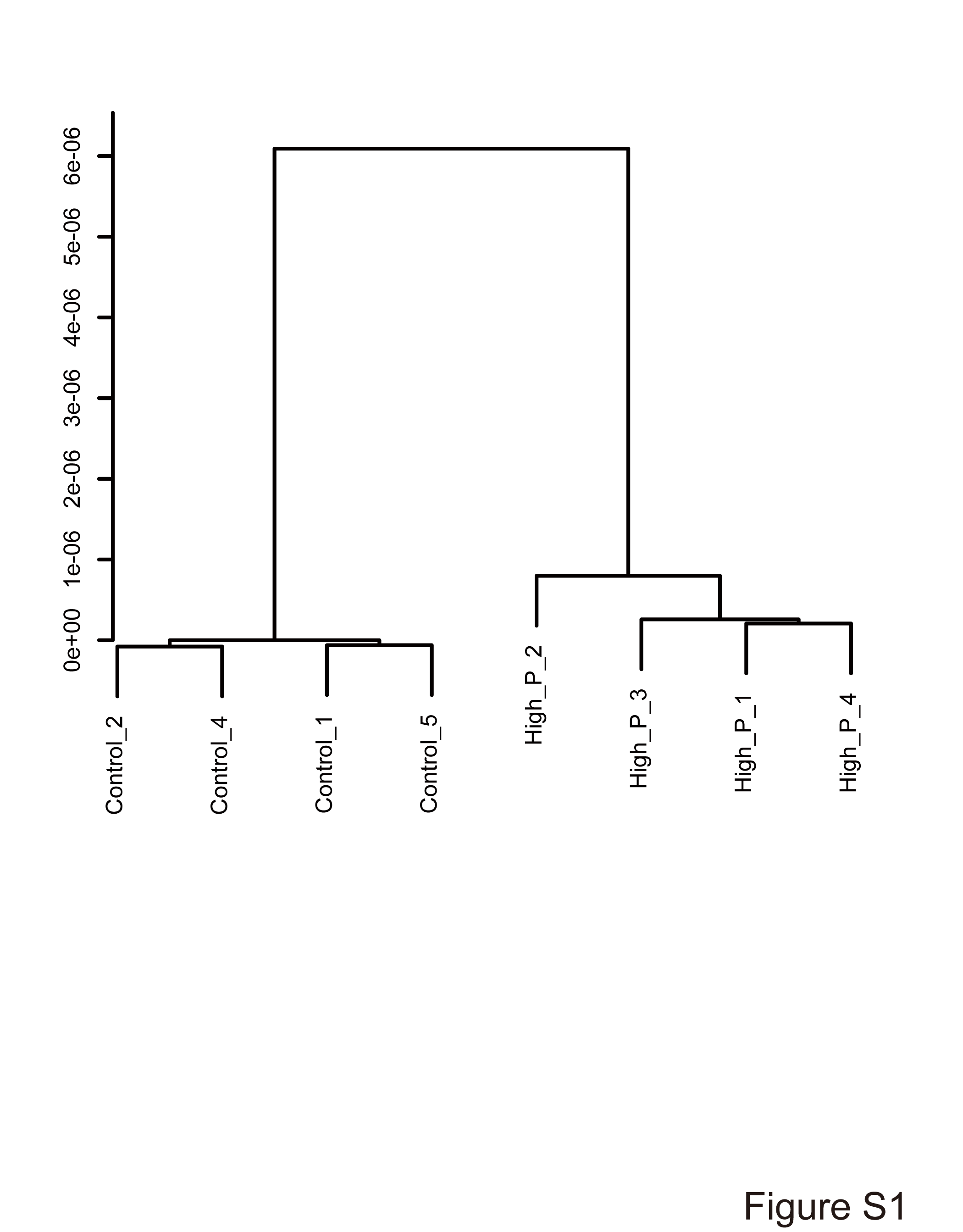

Supplement: Figure S1 — Hierarchical clustering dendrograms from DFW-quantified DNA microarray data. High_P, high phosphorus diet group. Numbers represent independent samples. The vertical scale represents between-cluster distances. (TIF) [file pone.0029483.s001.tif]
